# Photocrosslinking of Polyacrylamides Using [2 + 2] Photodimerisation of Monothiomaleimides

**DOI:** 10.1021/acs.macromol.2c01710

**Published:** 2022-09-29

**Authors:** Mohammed Aljuaid, Hannes A. Houck, Spyridon Efstathiou, David M. Haddleton, Paul Wilson

**Affiliations:** † Department of Chemistry, University of Warwick, Library Road, CoventryCV4 7AL, U.K.; ‡ Department of Chemistry, Turabah University College, Taif University, P.O. Box 11099, Taif21944, Saudi Arabia; § Institute of Advanced Study, University of Warwick, CoventryCV4 7AL, U.K.

## Abstract

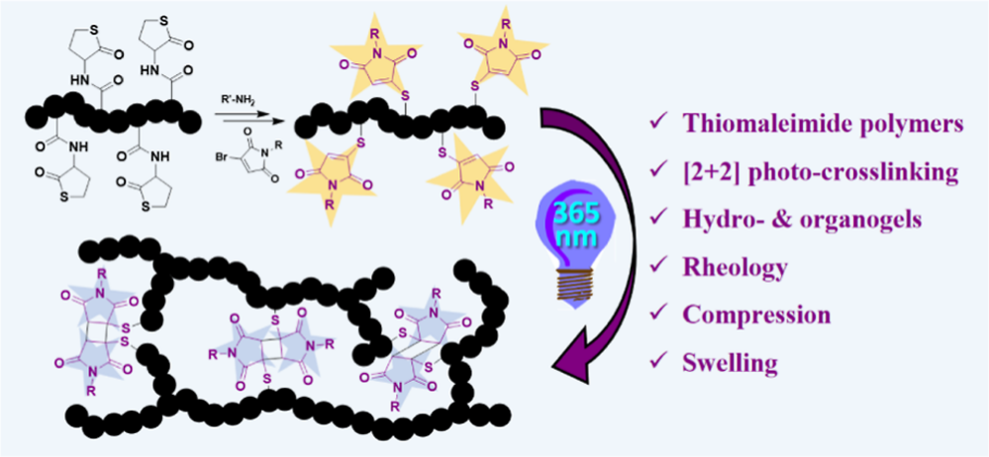

The [2 + 2] photocycloaddition of monothiomaleimides (MTMs) has been exploited for the photocrosslinking of polyacrylamides. Polymer scaffolds composed of dimethylacrylamide and varying amounts of d,l-homocysteine thiolactone acrylamide (5, 10, and 20 mol %) were synthesized via free-radical polymerization, whereby the latent thiol functionality was exploited to incorporate MTM motifs. Subsequent exposure to UV light (λ = 365 nm, 15 mW cm^–2^) triggered intermolecular crosslinking via the photodimerization of MTM side chains, thus resulting in the formation of polyacrylamide gels. The polymer scaffolds were characterized using Fourier transform infrared spectroscopy, UV–visible spectroscopy, ^1^H NMR spectroscopy, and size exclusion chromatography, confirming the occurrence of the [2 + 2] photocycloaddition between the MTM moieties. The mechanical and physical properties of the resulting gels containing various MTM mol % were evaluated by rheology, compression testing, and swelling experiments. In addition, scanning electron microscopy was used to characterize the xerogel morphology of 5 and 10 mol % MTM hydro- and organo-gels. The macro-porous morphology obtained for the hydrogels was attributed to phase separation due to the difference in solubility of the PDMA modified with thiolactone side chains, provided that a more homogeneous morphology was obtained when the photo-gels were prepared in DMF as the solvent.

## Introduction

Polymer scaffolds have proven to be useful materials for several applications, including tissue engineering,^1^ drug delivery,^2^ biosensors,^3^ and filtration membranes in separation processes for gaseous and liquid mixtures.^4^ These materials can be synthesized using several conjugation reactions, such as Diels–Alder,^5−8^ thiol–ene,^9−12^ and [2 + 2] photocycloaddition.^13−17^ Maleimides have been shown to be excellent candidates for these conjugation reactions due to the electrophilic characteristic that is attributed to the relatively low-energy π_C=C_* orbital. However, the [2 + 2] photocycloaddition of maleimides, in batch, is relatively slow, requiring long irradiation times and high-energy UV light (270–320 nm), which is not always desirable, especially for sensitive substrates such as biomolecules.^18^ Nonetheless, there have been several reports demonstrating the use of [2 + 2] photodimerization of maleimides for the formation of covalent crosslinked networks. For example, polymethacrylates bearing maleimide groups have been photochemically crosslinked under UV irradiation without the need for a photosensitizer,^19^ although a 2-fold increase in crosslinking rate occurred when thioxanthone was added. Another report introduced the photocrosslinking of both co- and ter-polyacrylamide copolymers using 2-(dimethylmaleimido)-*N*-ethyl acrylamide as the photoactive comonomer in order to obtain pH- and temperature-responsive hydrogels.^20^ It is noteworthy that incomplete curing of the maleimide side chain groups is typically achieved, although this can be exploited for further functionalization using Diels–Alder or thiol–ene reactions.^21,22^ This allows for the introduction of more complexity and functionality into the formed materials, for instance, by covalently attaching biomolecules to the scaffold using thiol–maleimide Michael addition. A two-photon-induced curing of maleimides using near-IR irradiation (λ = 800 nm) has also been developed, giving access to highly resolved [2 + 2]-crosslinked 3D microfabricated networks.^23^ Hence, an alternative [2 + 2]-crosslinking strategy for maleimide-based formulations at much longer wavelengths and lower energy became available, enabling a more benign route for hydrogel formation under physiological conditions.

**Figure 1:**
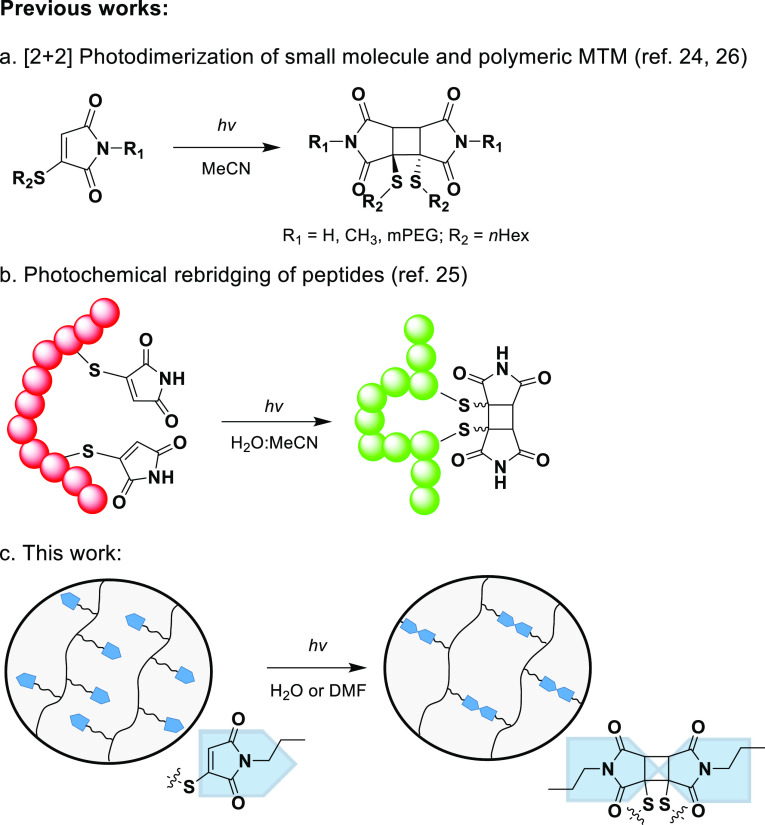
(a) Previous works on the [2 + 2] photocycloaddition of MTM for small molecule and polymer conjugation, (b) photochemical rebridging of MTM-modified biomolecules, and (c) exploitation of the MTM-[2 + 2] photocrosslinking for hydro- and organo-gel formation.

Recently, monothiomaleimide (MTM) has been reported as an efficient and highly specific reagent toward [2 + 2] photocycloaddition (Figure 1a).^24^ The substitution on the maleimide ring by a thiol group was shown to red-shift the wavelength of maximum absorption from 270 up to 339 nm. Moreover, MTMs have been demonstrated to undergo highly efficient and stereoselective (*exo*, head-to-head) [2 + 2] photodimerization within 5 min, including in water–acetonitrile mixtures (e.g., 95:5 v/v) at concentrations as low as 72 μM. This led to MTMs being excellent candidates for photochemically rebridging the disulfide bond in biomolecules (Figure 1b).^25^ Only recently, the potential of [2 + 2] photodimerization of MTMs was explored in polymer conjugation reactions, as illustrated by the quantitative coupling of linear and brush-like MTM end-capped hydrophilic polymers in only 10 min^26^

Herein, we further extend the scope of the thiolmaleimide [2 + 2] photodimerization reaction into polymer chemistry by reporting, for the first time, the synthesis of MTM side chain-functionalized polymers and their subsequent photocrosslinking under irradiation (Figure 1c).

## Results and Discussion

**Scheme 1:**
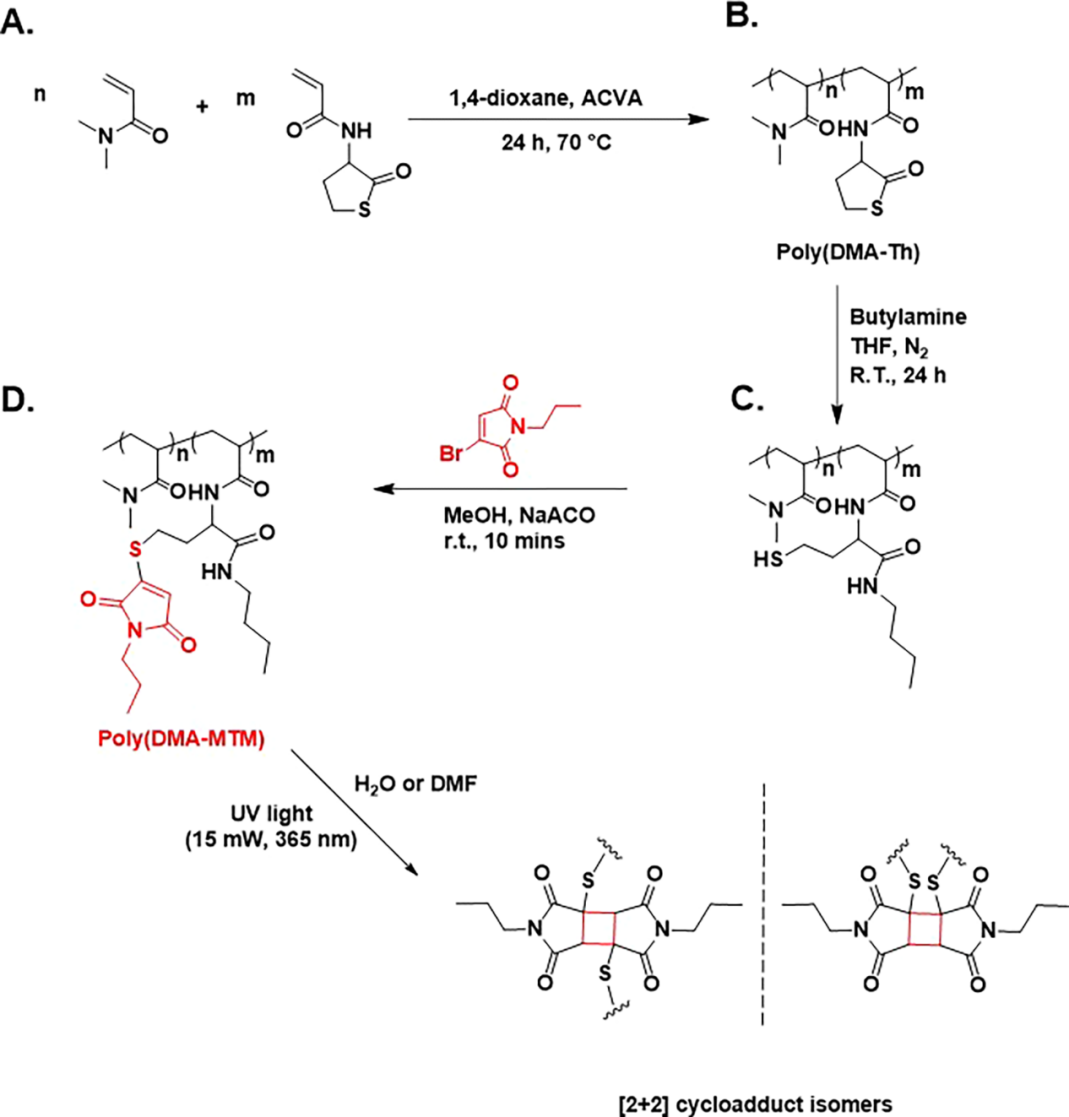
Schematic Representation of the Devised Synthesis Strategy toward poly(DMA-MTM) Scaffolds (A) Free-radical copolymerization of DMA and TLA, followed by (B) aminolysis of the side chains resulting in homocysteine thiolactone ring opening and the liberation of thiol functionalities. (C) Thiol–bromo post-modification reaction with MBM eventually gives the MTM functionalized polymers, (D) which can self-crosslink under UV irradiation via a [2 + 2] dimerization (365 nm, 15 mW cm^–2^).

In order to attach MTM into the polymer chain, the chosen strategy was to copolymerize dimethyl acrylamide (DMA) and d,l-homocysteine thiolactone acrylamide monomer (TLA) via free-radical polymerization (FRP) (Scheme 1A). The resulting TLA-containing polymer precursor hence provided an alternative route for the incorporation of the MTM motifs into the polymer scaffolds via a straightforward post-polymerization reaction. This strategy was devised since direct use of an MTM–acrylamide monomer was expected to result in side reactions during its FRP, which is commonly observed for pendant maleimide motifs. As previously reported, TLA readily undergoes ring opening via aminolysis, thereby liberating a free sulfhydryl group that becomes available for further functionalization (Scheme 1B).^27^ In our approach, these free sulfhydryls were used to react with *N*-propyl monobromomaleimide (MBM) via an addition–elimination mechanism to obtain the MTM-functionalized polymer scaffolds (Scheme 1C). In a final step, the MTM-based polymer solutions were exposed to UV light (365 nm, 15 mW cm^–2^), leading to the [2 + 2] photodimerization of MTM moieties and thus crosslinking of the polymer (Scheme 1D).

The synthesis of TLA monomer was achieved by the addition of acryloyl chloride to d,l-homocysteine thiolactone hydrochloride according to a literature procedure.^28^ The monomer was characterized by ^1^H and ^13^C NMR (Figure S1, Supporting Information) with the most important finding for the TLA monomer characterization being the presence of the olefin group at 5.65 and 6.18 ppm, thus indicating the successful synthesis of the monomer. Additionally, the N–H peak of the amide group was observed at 8.46 ppm, which confirmed the formation of the acrylamide monomer. The integration of all proton signals agreed with the chemical structure of the monomer.

The synthesis of MBM compound was achieved by reacting propylamine with bromomaleic anhydride. ^1^H and ^13^C NMR spectroscopy confirmed the chemical structure of MBM (Figure S2, Supporting Information). Indeed, the methylene peak directly linked to the imide group was detected at 3.45 ppm, and its integration with the olefin peak at 6.8 ppm was found to be 2:1, respectively. Electron-spray-ionization mass spectroscopy confirmed the molecular weight of MBM, and the presence of bromine atom isotopic distribution was evident. This finding was important to ensure that the MBM compound could participate in the addition–elimination reaction with the latent sulfhydryl groups from the polymer scaffolds.

**Figure 2:**
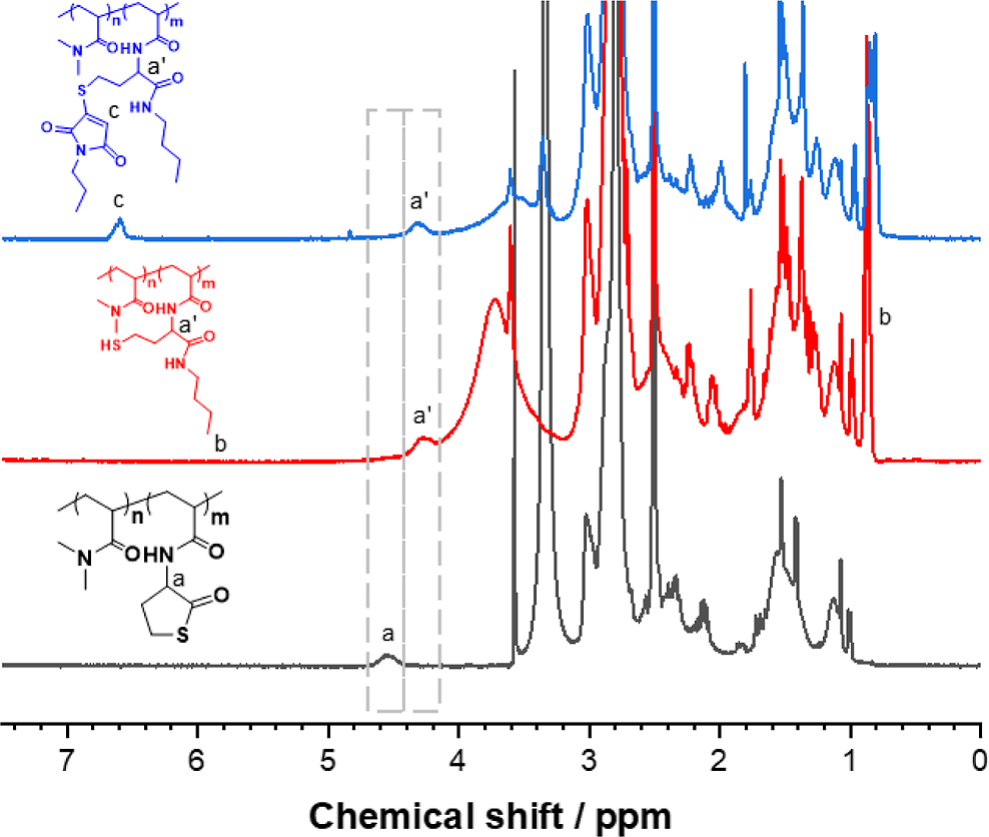
^1^H NMR spectra of poly(DMA-TLA)_10%_ (black, bottom), the ring-opened poly(DMA-TLA)_10%_ (red, middle), and poly(DMA-MTM)_10%_ (blue, top).

The TLA monomer was copolymerized with DMA, which was selected as a hydrophilic acrylamide monomer. Three copolymers were synthesized by FRP in 1,4-dioxane using 4,4′-azobis(4-cyanovaleric acid) (ACVA) as an initiator with different comonomer compositions through changing the relative mole fraction of DMA and TLA (Table 1). The polymerization was performed at 70 °C and left overnight with monomer conversion determined by ^1^H NMR. The TLA content was calculated by integrating the dimethyl peak of DMA (2.65–3.09 ppm) and the C–H of TLA (4.55 ppm) (Figure 2A). Table 1 summarizes the experimentally observed TLA composition and the molecular weight data and dispersity obtained from size exclusion chromatography (SEC) measurements of the prepared copolymers (Figure S4).

**Table 1:** Characterization of the Synthesised Poly(DMA-TLA) Copolymers

entry	TLA %^a^	*M*_w_,SEC (g·mol^–1^)^b^	*M*_n_,SEC (g·mol^–1^)^b^	*D̵*
poly(DMA-TLA)5%	5.3	62,400	22,100	2.82
poly(DMA-TLA)10%	9.8	64,200	19,200	3.33
poly(DMA-TLA)20%	25	62,100	21,400	2.89

a TLA % was calculated by the integration ratio between DMA (δ = 2.65–3.09 ppm) and the CH of the TLA peak (δ = 4.55 ppm).

b Determined from SEC analysis using narrow PMMA standards.

Subsequently, the thiolactone moieties were ring-opened through aminolysis with *n-*butylamine in order to release the latent thiol functionality (Figures 2, S3, and S5). The proton resonances corresponding to TLA could be used to evaluate the ring-opening step as the signal of CH from the thiolactone ring would be shifted from 4.55 to 4.27 ppm. Additionally, the methyl group from *n*-butylamine was observed at 0.87 ppm. Eventually, MBM was introduced to react with the liberated sulfhydryl functionalities which resulted in the formation of poly(DMA-MTM)s bearing thiolmaleimide groups in the polymer side chains (Figures 2 and S7). The successful incorporation of MTM in the polymer scaffolds was confirmed by ^1^H NMR as the olefin proton appeared at 6.60 ppm. The sulfhydryl degree of modification was calculated from the ratio of the C–H peak at 4.27 ppm and the olefin peak at 6.60 ppm and was found to be 90, 95, and 75% for poly(DMA-TLA)_5%_, poly(DMA-TLA)_10%_, and poly(DMA-TLA)_20%_, respectively. It is possible that some disulfide bond formation occurred during the aminolysis process, explaining why the sulfhydryls did not reach full conversion to MTMs.

**Figure 3:**
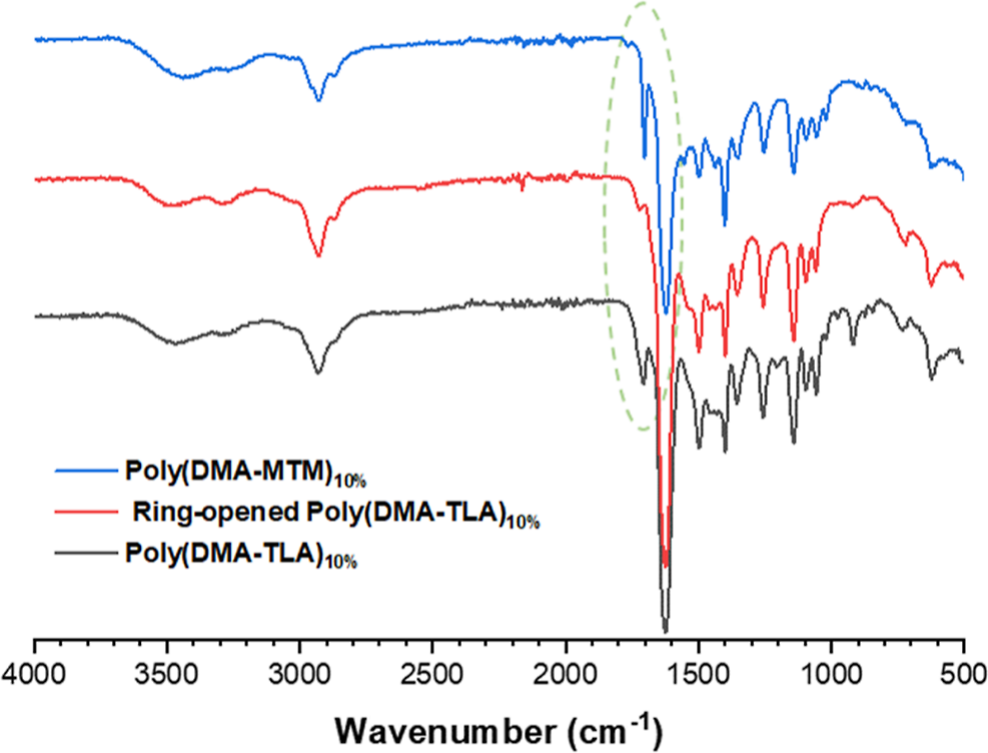
FT-IR spectroscopy of poly(DMA-TLA)_10%_ (black, bottom), the ring-opened poly(DMA-TLA)_10%_ (red, middle), and poly(DMA-MTM)_10%_ (blue, top).

The sequential polymer modification steps were additionally analyzed by FT-IR spectroscopy to further evidence the thiolactone ring opening and the formation of sulfhydryl groups, as observed by the significant reduction of the peak at 1705 cm^–1^ after the aminolysis step. The covalent attachment of MTM onto the polymer was also confirmed by FT-IR spectroscopy, with an increase in the peak at 1705 cm^–1^ attributed to the introduction of the imide group from MTM (Figures 3,S9, and 10).

Besides NMR and IR spectroscopy, SEC analysis was also performed to monitor the polymer modification steps via the traces arising from the refractive index (RI, Figures S6 and S8) and UV detectors. The UV detector was set at λ = 365 nm, selected based on the absorption spectra of the poly(DMA-MTM) scaffolds (Figure S11B,D,F) which exhibited little absorbance above 360 nm prior to reaction with MBM. Pleasingly, the traces from the RI and UV coincided, thereby confirming the covalent attachment of MTMs to the polymer and thus that the targeted scaffolds had been successfully synthesized (Figure S11A,C,E).

**Figure 4:**
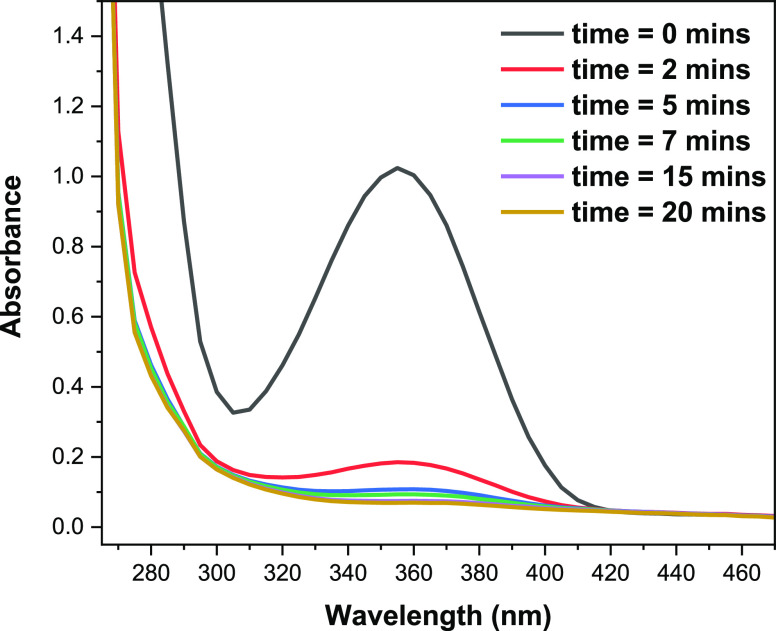
UV–vis spectroscopy monitoring the consumption of MTMs (λ = 360 nm) during the irradiation of poly(DMA-MTM)_10%_ solution using a 365 nm Luminox II 96-position LED array set at 45 mW (15 mW cm^–2^).

With the MTM-functionalized polymer scaffolds in hand, their potential to undergo photocrosslinking to yield polyacrylamide gels was next explored. At first, the photoreactivity of the MTM-containing polymers was investigated. For this, UV–vis spectroscopy was used to monitor the chemical changes occurring during the UV irradiation. The absorbance peak for MTM, at 360 nm, significantly reduced after only 2 min and continued to reduce in intensity until complete consumption was observed after 20 min (Figure 4). This experiment was conducted as additional proof of the occurrence of the MTM-[2 + 2] photocycloaddition being responsible for the photogelation.

**Figure 5:**
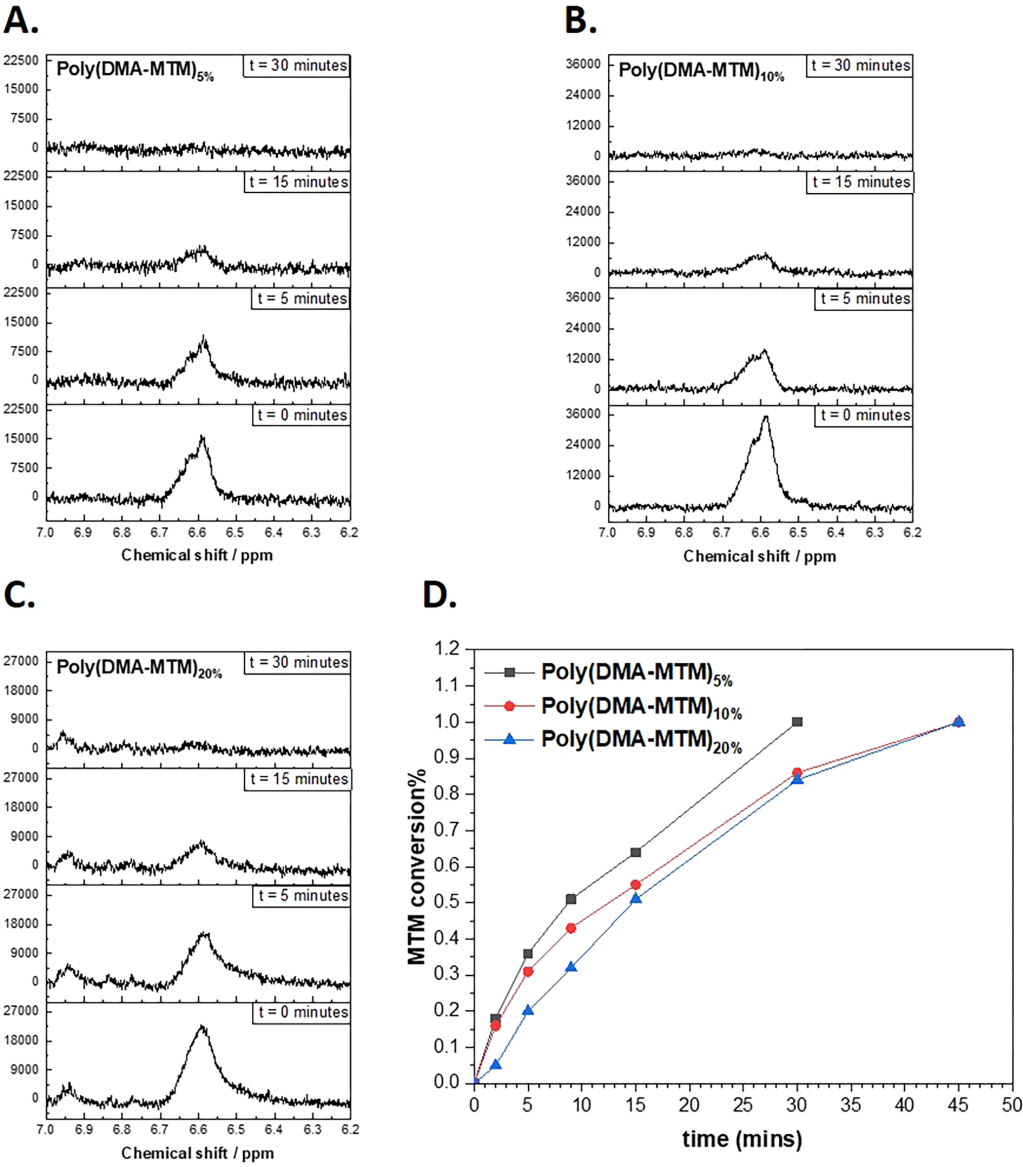
Olefinic peak followed during the irradiation for (A) poly(DMA-MTM)_5%_, (B) poly(DMA-MTM)_10%_, and (C) poly(DMA-MTM)_20%_; (D) kinetics of the [2 + 2] photodimerization of MTM within the polymers; the *y*-axis represents the following olefinic peak collected by ^1^H NMR in DMSO-*d*_6_, and the *x*-axis represents the irradiation time.

Besides UV–vis monitoring, additional experiments were performed using ^1^H NMR spectroscopy. For this, the poly(DMA-MTM) polymers were dissolved in DMSO-*d*_6_ at low concentrations (i.e., 2 wt %) and exposed to UV light (365 nm, 15 mW cm^–2^) in order to follow the chemical changes of the MTM moieties under irradiation. Although these reaction conditions did not lead to gelation, valuable information about the reaction kinetics of the macromolecular substrates was obtained. The olefin peak at 6.60 ppm was monitored by ^1^H NMR over time during UV exposure, which decreased during the irradiation time until it was fully consumed within 45 min (Figure 5A–C). Plotting the conversion against the irradiation time revealed that the consumption of MTM was of the order of poly(DMA-MTM)_5%_ > poly(DMA-MTM)_10%_ > poly(DMA-MTM)_20%_, with poly(DMA-MTM)_5%_ reaching 100% conversion after 30 min, while poly(DMA-MTM)_10%_ and poly(DMA-MTM)_20%_ reached 85 and 80%, respectively. However, both poly(DMA-MTM)_10%_ and poly(DMA-MTM)_20%_ reached near-quantitative conversion after 45 min of UV irradiation (Figure 5D).

**Figure 6:**
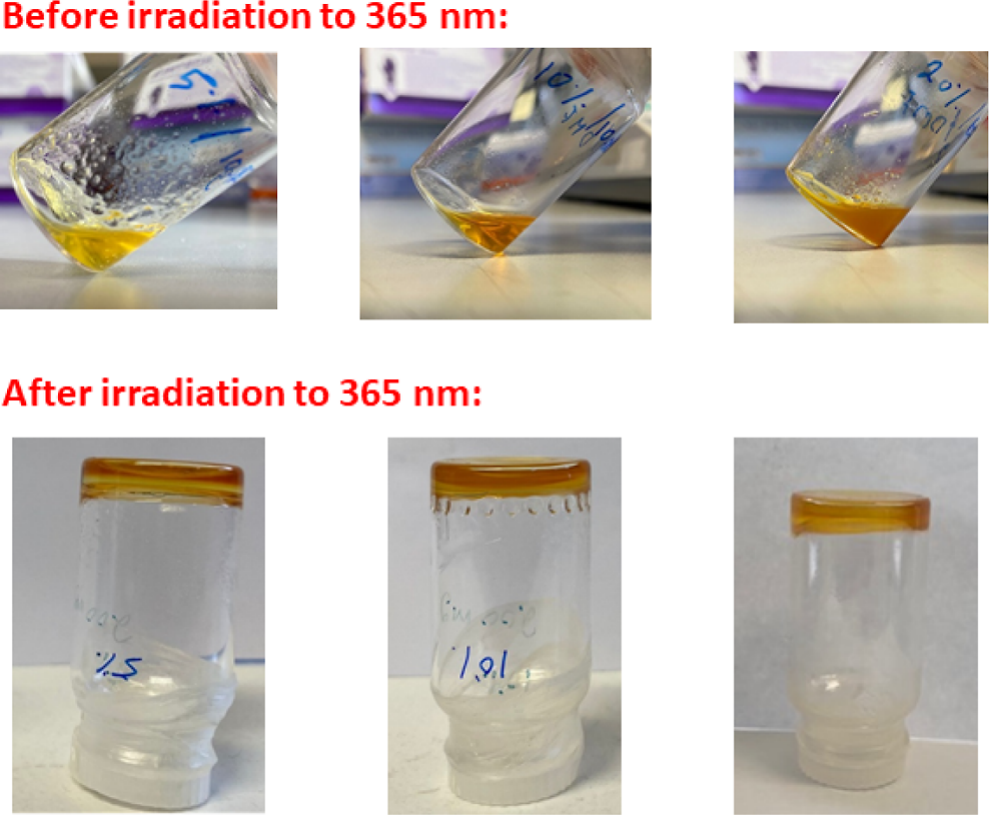
Pictures of the poly(DMA-MTM)_5%_ (left), poly(DMA-MTM)_10%_ (middle), and poly(DMA-MTM)_20%_ (right) gels before (top) and after (bottom) 48 h of UV irradiation (365 nm, 15 mW cm^–2^) in DMF.

Having confirmed the photoreactivity of the MTM moieties, the poly(DMA-MTM) substrates were then dispersed in aqueous media to examine their photocrosslinking for the formation of hydrogels. While the 5 and 10% functionalized PDMA polymers remained water-soluble, polymers containing 20% of MTM were insoluble in water. Consequently, for the purposes of gelation, the solvent was changed to DMF, which was shown to be a good solvent for all the scaffolds under investigation. Different concentrations were tested in order to select the appropriate condition for gelation. It was observed that 20 wt % of poly(DMA-MTM)_20%_ was the solubility limit; therefore, this concentration was chosen for all the scaffolds in order to investigate the photocrosslinking. Thus, 20 wt % of the three polymer scaffolds were dissolved in DMF and the solutions were exposed to UV light (365 nm, 15 mW cm^–2^) for 48 h to ensure complete conversion. Gels were qualitatively formed from each polymer solution as shown by a simple vial inversion test (Figure 6). The photo-gels were then dried in a vacuum oven in order to remove the DMF solvent and then characterized using FT-IR spectroscopy. The aim for IR characterization was to assess whether photocrosslinking indeed occurred due to [2 + 2] photodimerization of MTM. This was confirmed as the imide group from the thiolmaleimide ring is expected to have a lower wavenumber due to the conjugation between the imide and C=C, which is lost upon the formation of the succinimide cycloadducts after the photodimerization, thus leading to a small increase in the imide wavenumber. FT-IR revealed that the imide group of the uncured polymer appeared at 1702 cm^–1^ but shifted to 1712 cm^–1^ after the gelation (Figure S13, Supporting Information).

**Figure 7:**
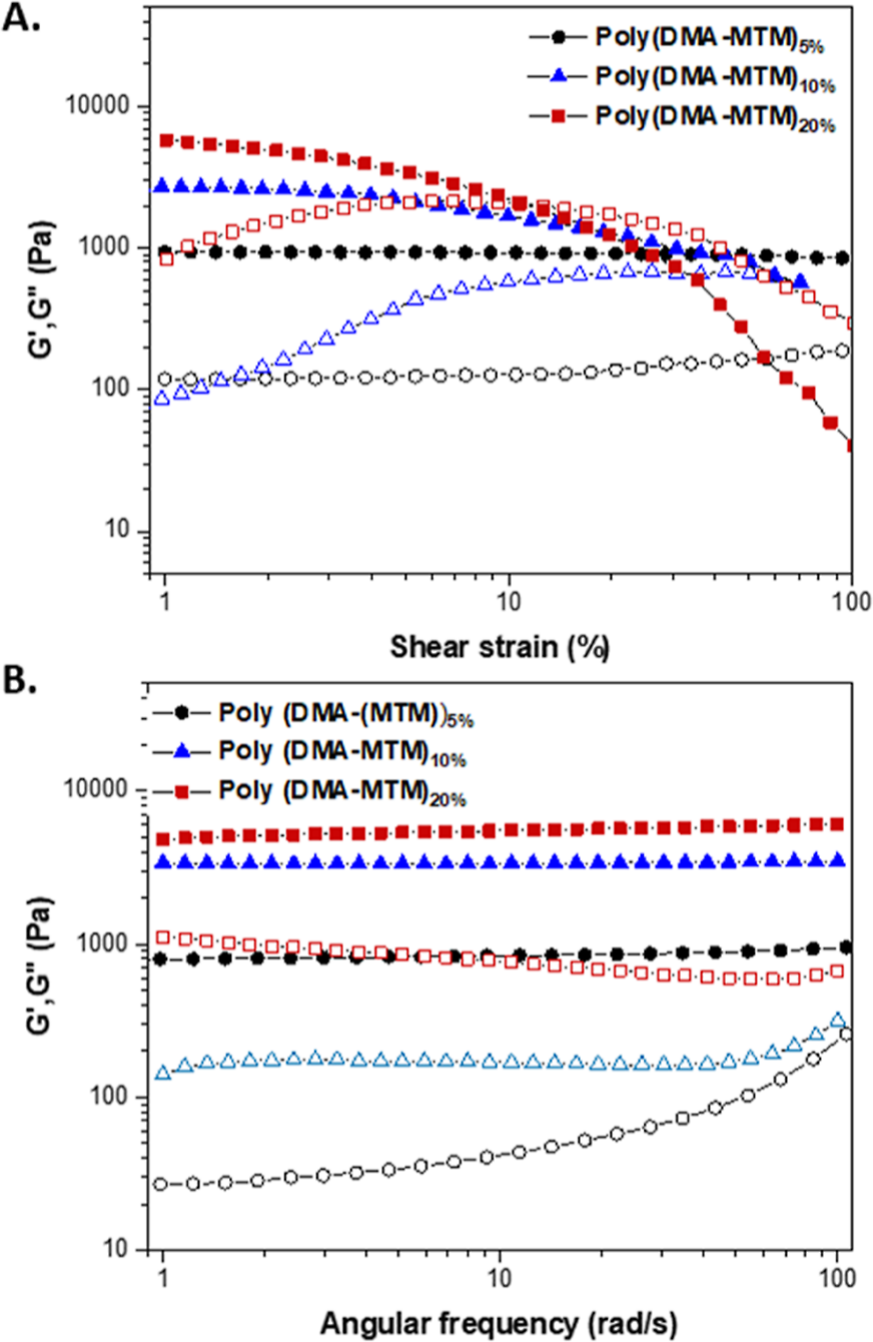
Amplitude sweep measurements of poly(DMA-MTM)s photo-gels at a constant frequency of ω = 10 rad·s^–1^ at 25 °C (A) and frequency sweep measurements of poly(DMA-MTM)s photo-gels using a constant strain of γ = 1% at 25 °C (B).

With successful photocrosslinking demonstrated, the viscoelastic properties of the photo-cured gels were evaluated by oscillatory shear mode rheology at 25 °C, allowing for their storage (*G*′) and loss (*G*″) moduli to be determined. Amplitude sweep experiments at a constant frequency of 10 rad s^–1^ were carried out on all samples to confirm that all measurements were conducted within the linear viscoelastic regime (Figure 7A). The *G*′ value of the photo-gel that contained the highest amount of MTM moieties [i.e., poly(DMA-MTM)_20%_] was higher than that observed for poly(DMA-MTM)_10%_ and poly(DMA-MTM)_5%_, respectively, indicative of a higher crosslinking density. The *G*′ values for poly(DMA-MTM)_5%_ were constant during the measurement region (1–100%), while poly(DMA-MTM)_20%_ and poly(DMA-MTM)_10%_ reached a crossover point at 11 and 71% shear strains, respectively, showing that a higher MTM content turned the materials more brittle preventing them from withstanding high shear strains along with a faster decrease in their *G*′. Next, frequency sweep experiments were conducted at 25 °C and at a constant strain value (γ) of 1%, selected based on the amplitude sweeps wherein *G*′ was fairly constant for all the photo-gels. The angular frequency was altered from 1 to 100 rad s^–1^ (Figure 7B). It was seen that the *G*′ was constant for all the materials during the experiment regardless of the photocrosslinking density, indicating the formation of a stable covalent network.^29^ The highest *G*′ value was found for poly(DMA-MTM)_20%_ (6000 Pa), while 900 and 2800 Pa were found for poly(DMA-MTM)_5%_ and poly(DMA-MTM)_10%_, respectively (Table S1). A similar trend was observed for the *G*″ with 5% MTM showing the lowest value (144 Pa), while the 20% MTM had the highest *G*″ value (800 Pa).

**Figure 8:**
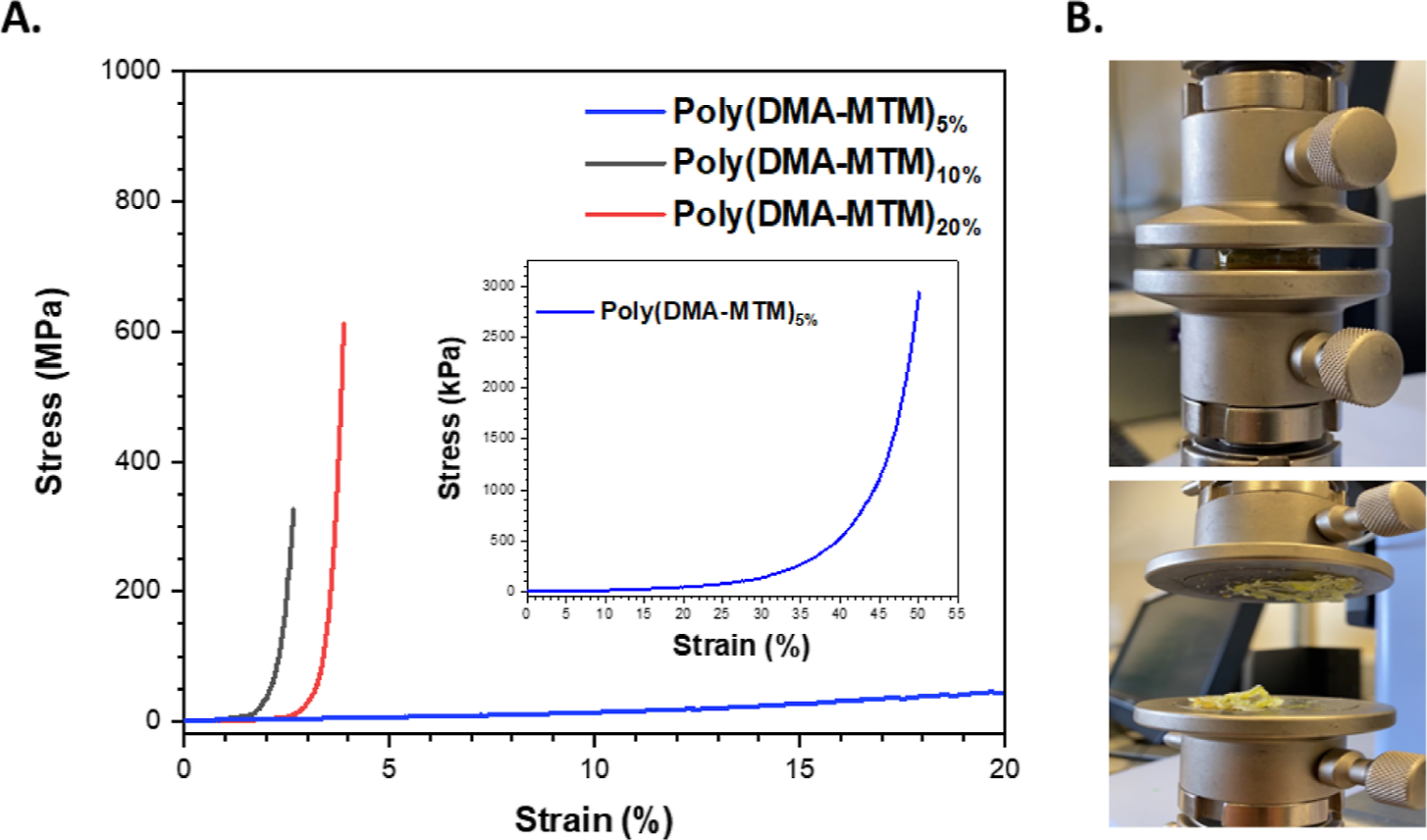
Compression test for poly(DMA-MTM)_5%_, poly(DMA-MTM)_10%_, and poly(DMA-MTM)_20%_ gels (A); a photo-gel before and after the compression test experiment (B).

Complementary to rheology investigations, compression tests were performed in order to characterize their elasticity and resistance against force. These experiments were performed since the poly(DMA-MTM)_20%_- and poly(DMA-MTM)_10%_-based gels were noticed to be very brittle, while their poly(DMA-MTM)_5%_ analogue seemed to be resistant against deformation and more flexible under pressure. These visual observations were confirmed by compression test measurements and revealed that a higher photocrosslinking density resulted in a higher stress point (Figure 8A). The actual value of poly(DMA-MTM)_5%_ stress was considerably low in comparison to the other two photo-gels (i.e., 2.9 MPa). There was no significant difference in the strain values of poly(DMA-MTM)_10%_ and poly(DMA-MTM)_20%_, and they were found in between 2.5 and 3.5%, which explained the brittle characteristics of these photo-gels. However, poly(DMA-MTM)_20%_ was found to be capable of withstanding more stress than poly(DMA-MTM)_10%_ as expected (Figure 8A). The brittleness of poly(DMA-MTM)_10%_ and poly(DMA-MTM)_20%_ could be attributed to the high photocrosslinking density and the increase in the heterogeneity of the photogels, thereby restricting polymer chain extension when stress was applied.^29^

**Figure 9:**
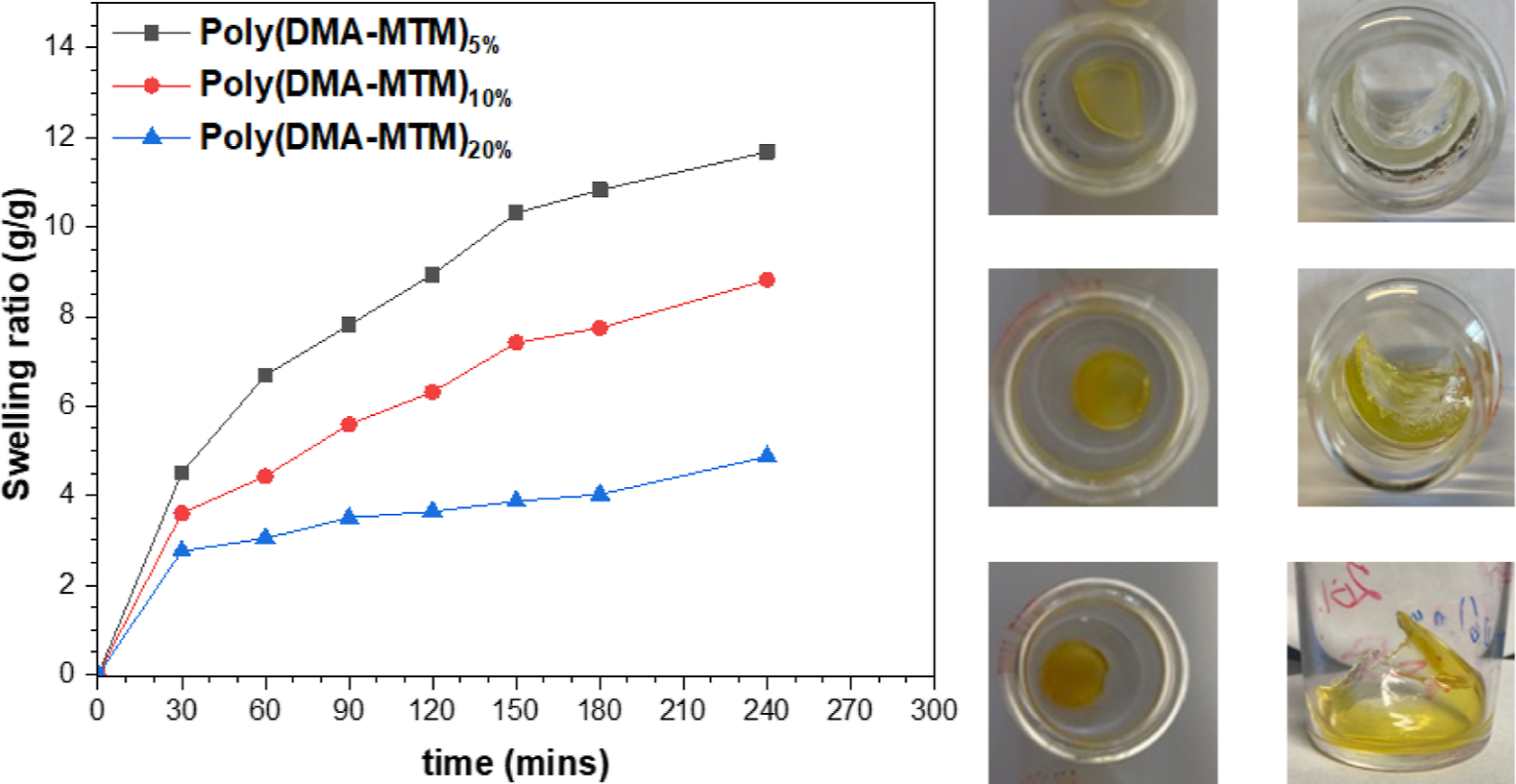
Swelling test for poly(DMA-MTM)_5%_, poly(DMA-MTM)_10%_, and poly(DMA-MTM)_20%_ xerogels; the *y*-axis represents the swelling ratio determined from the weight of the swollen and xerogel; the *x*-axis represents the swelling period from 0 to 240 min.

Finally, swelling tests were performed in water to investigate the difference in the swelling behavior of the xerogels, obtained after vacuum drying (Figure 9). Theoretically, the lower the crosslink density, the higher the solvent amount that can be taken up by the xerogel.^30^ Plotting the swelling ratio, which was calculated from eq 1, against time revealed that poly(DMA-MTM)_5%_ swelled more in water (12 g/g, 240 min) compared to poly(DMA-MTM)_10%_ (9 g/g, 240 min) and poly(DMA-MTM)_20%_ (5 g/g, 240 min) due to the formation of a bigger mesh size able to occupy a higher amount of water, agreeing well with the low *G*′ values. The final equilibrium swelling ratio was measured after 48 and 72 h and found to be 15, 10, and 6 g/g for poly(DMA-MTM)_5%_, poly(DMA-MTM)_10%_, and poly(DMA-MTM)_20%_, respectively
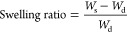
1where *W*_d_ and *W*_s_ are the weights of the dried and swollen gels, respectively.

**Figure 10:**
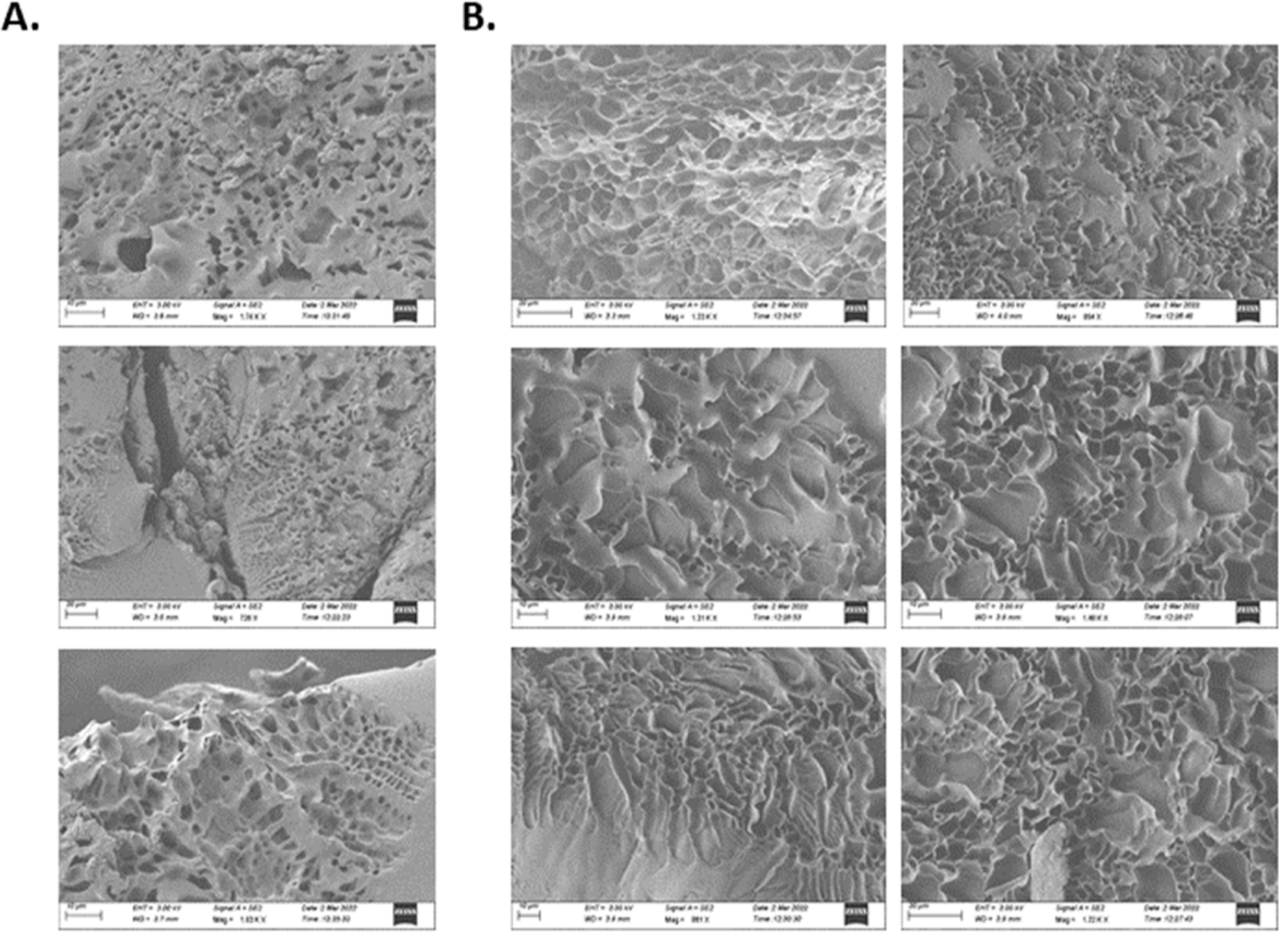
SEM of dried poly(DMA-MTM)_5%_ (A) and poly(DMA-MTM)_10%_ (B), prepared from water. The size of the scale bar is 10 μm.

Since poly(DMA-MTM)_20%_ was insoluble in water leading to undesirable precipitation (Figure S14, Supporting Information), only poly(DMA-MTM)_5%_ and poly(DMA-MTM)_10%_ were formulated in aqueous media and their inner structure was visualized by scanning electron microscopy (SEM). It was observed that both xerogels had porous morphologies with poly(DMA-MTM)_10%_, demonstrating larger pores (11.19 μm) than poly(DMA-MTM)_5%_ (2.64 μm) (Figure 10). Initially, this was surprising considering that a lower crosslinking density should result in larger mesh sizes within the network matrix. However, having previously hypothesized that increasing the mole fraction of the MTM-functionalized side chains reduced the water solubility of the scaffolds, in combination with the observed macro-phase separation of poly(DMA-MTM)_20%_ during swelling in water, the increase in pore size here was likely attributed to the MTM-functionalized side chains arranging themselves to minimize their interactions with water, thereby leading to larger pores. In contrast, no macro-porosity was observed from the SEM images of poly(DMA-MTM)_5%_ and poly(DMA-MTM)_10%_ xerogels synthesized in DMF (Figures S15 and S16, Supporting Information), reinforcing this theory. In fact, the xerogels formed using DMF solvent seemed to be homogeneous with an absence of phase separation as explained by the better solubility of the polymer precursors in the organic solvent. These data indicated the presence of phase separation when water solvent was used, which could be useful for some applications that require such macro-porous networks, such as biomedical applications and tissue engineering.^31^

## Conclusions

In conclusion, MTM-based polyacrylamide copolymers were synthesized and transformed into their corresponding gels after exposing their solutions to UV light (λ = 365 nm). Depending on the crosslink density, these materials exhibited different mechanical and physical properties, which were attributed to the difference in MTM content incorporated into the polymer precursors. The 10 and 20% MTM were found to be brittle materials, while the 5% MTM material appeared flexible and highly stretchable. The swelling behavior demonstrated the inversely proportional relationship between the crosslink density and the amount of water absorbed by the xerogels. The rheological characterization further evidenced the direct effect of the MTM content on the storage and loss moduli in amplitude and frequency sweep experiments. The photo-gels were formulated in both aqueous and organic solvents and investigated using SEM, showing that the hydrogels of 5 and 10% MTM exhibited phase separation, which resulted in the formation of macro-porous materials. On the other hand, the organo-gels did not form any macro-pores and appeared to be well packed. This report introduced the first method of synthesizing MTM-functional polymers that can be transformed into covalent networks under UV light via the [2 + 2] photocycloaddition of MTMs. Although the strategy used to synthesize these scaffolds involves a multi-step process, it provides a broad scope to access different photocurable materials with ranging properties by changing the bromomaleimide *N*-substituent and/or amine used in the thiolactone ring opening.^32^

## Notes

Raw data files for the figures presented in the manuscript have been deposited in a local open access repository at Warwick (https://wrap.warwick.ac.uk/) and are also available on request.

## Supporting Information Available

Experimental procedures, instrumentation, synthetic procedures, and supporting characterization of the synthesized materials (PDF)
