# Supplementary material for: Photocrosslinking of Polyacrylamides Using [2 + 2] Photodimerisation of Monothiomaleimides
Source: Macromolecules. 2022 Sep 29;55(19):8495–504. doi: 10.1021/acs.macromol.2c01710 (PMC9558485; doi:10.1021/acs.macromol.2c01710)
Supplement: Supplementary file 1 — ma2c01710_si_001.pdf [file ma2c01710_si_001.pdf]

Supporting Information

Photocrosslinking of polyacrylamides using [2+2] photodimerisation of monothiomaleimides

Mohammed Aljuaid,[a,b] Hannes A. Houck ,[a,c] Spyridon Efstathiou,[a] David M. Haddleton [a*] Paul Wilson [a*]

[a] *Department of Chemistry, University of Warwick, Library Road, CV4 7AL, Coventry, UK.*

[b] *Department of Chemistry, Turabah University College, Taif University, P.O. Box 11099, Taif 21944, Saudi Arabia.*

[c] *Institute of Advanced Study, University of Warwick, CV4 7AL, Coventry, UK.*

E-mail: p.wilson.1@warwick.ac.uk; d.m.haddleton@warwick.ac.uk

Experimental

1. Materials

All reagents were used as received from their suppliers without further purification unless stated otherwise. Bromomaleic anhydride 97%, DL-Homocysteine thiolactone hydrochloride 99%, sodium hydrogen carbonate, sodium acetate anhydrous were purchased from Fisher Scientific. Propylamine 99%, 1, 4-dioxane >99%, *N,N*-dimethylacrylamide 99% were purchased from Aldrich. Acryloyl chloride with 400 ppm phenothiazine, 4,4'-azobis(4-cyanovaleric acid) 98% were purchased from ALFA. Deuterated solvents (CD_3Cl, and DMSO) were purchased from Aldrich.

2. Instruments

The instruments used for all experiments were Infrared Bruker vector 22 FT-IR spectrometer provided by a golden gate diamond attenuated reflection cell, and the samples were run from 550 – 4000 cm^{-1} wavenumber.

^{1}H and ^{13}C NMR spectra were obtained using Bruker AV-300, HD-300, and HD-400 spectrometers utilising deuterated solvents provided from Sigma-Aldrich. Chemical shifts are given in ppm downfield from the internal standard tetramethylsilane.

UV-Vis spectra were recorded on an Agilent Technologies Cary 60 UV-Vis in the range of 200-800 nm using a cuvette with a 10 mm optical path length.

Mass spectrometry was conducted using Agilent 6130B ESI-Quad containing an autosampler and isocratic pump from an Agilent 1100 to deliver the samples and the solvent used. In this system, the solvent was 80:20 methanol: water (HPLC grade).

Size exclusion chromatography was performed using Agilent Infinity II MDS instruments equipped with differential refractive index (DRI), viscometry (VS), dual angle light scatter (LS) and multiple wavelength UV detectors. The system was equipped with 2 x PLgel Mixed C columns (300 x 7.5 mm) and a PLgel 5 µm guard column. The eluent used was DMF with a 5 mmol NH_4BF_4 additive to reduce column interactions. Samples were run at 1 ml/min at 50 °C. Poly(methyl methacrylate) standards (Agilent EasiVials) were used for calibration between 955,000 – 550 g/mol. Analyte samples were filtered through a GVHP membrane with 0.22 µm pore size before injection. Respectively, experimental number average molecular weights ($M_{n, SEC}$) and dispersity ($Đ$) values of synthesized polymers were determined by conventional calibration using Agilent GPC/SEC software.

[2+2]-photocycloaddition reactions were performed using a 365 nm Lumidox II 96-Position LED array set at 45 mW. Irradiance (mW cm^{-2}) was estimated by using an S142C Integrating Sphere Power Sensor Head with Silicon Detector, connected to a ThorLabs PM400 optical power meter at a wavelength set to 365 nm.

Rheological analysis was performed in an Anton Paar MCR 302 rheometer equipped with a parallel plate configuration (25 mm diameter). The data was analysed using the RheoCompass software.

Compression tests were carried out using a Shimadzu EZ-LX Universal Testing Instrument equipped with a 50 N load cell.

Scanning Electron Microscopy (SEM) was conducted using Zeiss SUPRA 55VP-23-99 scanning electron microscope (SEM) with a field emission electron gun (FEG). Accelerating voltage used was at 5 kV and

both SE2 and nLens detectors were utilised depending on the sample. Unless otherwise stated, hydrogels were frozen in liquid nitrogen and freeze dried to remove water. Freeze dried gels were then casted on carbon tabs (9 mm) attached to aluminium specimen stubs. To improve the sample imaging, gold (Au) sputter coating was applied for 30 seconds prior imaging.

3. Synthetic procedures

Homocysteine thiolactone acrylamide monomer (TLA)

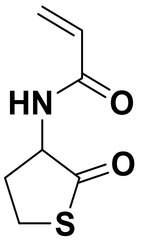

The reaction was adopted from previous literature.[1] DL-homocysteine thiolactone hydrochloride (14 g, 91 mmol) was dissolved in 100 mL 1,4-dioxane and 100 mL water. Sodium hydrogen carbonate (76.5 g, 910 mmol) was added to the solution. After immersing the solution flask in an ice bath, the reaction was allowed to reach 0 °C and acryloyl chloride (9 g, 100 mmol) was added dropwise for 30 minutes and the reaction was carried out overnight. 200 mL water was added to the reaction mixture and the solution was extracted by ethyl acetate (3 x 200 mL). The organic layer was collected and the solvent removed under reduced pressure. The product was then recrystallized from dichloromethane to obtain the title compound as a white solid in 70 % yield (10.9 g, 63.7 mmol). ^{1}H NMR (300 MHz, DMSO-d_6) δ (ppm) 2.1 (m, 1 H), 2.45 (m, 1 H), 3.36 (m, 2 H), 4.70 (m, 1 H), 5.59 - 5.72 (m, 1 H), 6.20 (m, 2 H), 8.46 (d, *J*=8.07 Hz, 1 H); ^{13}C NMR (75 MHz,DMSO-d_6) δ (ppm) 27.33, 30.74, 58.66, 126.65, 131.53, 165.10, 205.80.

***N*-Propyl-3-bromomaleimide**

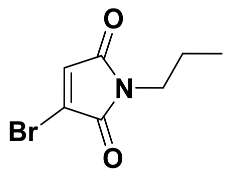

The reaction was adopted from previous literature.[2] Bromomaleic anhydride (8.0 g, 45.4 mmol) was dissolved in acetic acid (200 mL) and propylamine (3.8 mL, 45.4 mmol) was slowly added to the solution. The reaction mixture was stirred and heated at 130 °C for 6 hours. Acetic acid was removed by the addition of toluene followed by azeotropic distillation, which was repeated several times. The crude product was purified by dry-loading flash chromatography using (70 % petroleum ether and 30 % ethyl acetate) to obtain *N*-propyl-3-bromomaleimide as a brown solid in 75 % yield (7.3 g, 33.6 mmol). IR υ/cm^{-1} 3100, 2950, 2125, 1692, 1400, 550-600; ^{1}H NMR (400 MHz, $CDCl_3$) δ (ppm) 0.83 (t, J = 7.4 Hz, 3H), 1.56 (s, J = 7.3 Hz, 2H), 3.45 (t, J = 7.2 Hz, 2H) 6.8 (s,1H); ^{13}C NMR (75 MHz, $CDCl_3$) δ (ppm) 11, 22, 40, 131, 132, 165, and 168); (MS) m/z 271.96 $([C_8H_{12}Br^{79}NNaO_3]^+)_{th}$ 272.22 $([C_8H_8Br^{79}NNaO_3]^+)_{exp}$, 273.96 $([C_8H_8Br^{81}NNaO_3]^+)_{th}$, 274.21 $([C_8H_8Br^{81}NNaO_3]^+)_{exp}$ (M^+: M^{2+} = 1 :1).

General procedure for the copolymerisation of dimethyl acrylamide (DMA) and homocysteine thiolactone (TLA) monomers

N,*N*-dimethylacrylamide and homocysteine thiolactone acrylamide (20 wt%) were dissolved in 1,4-dioxane (200 mL), followed by the addition of 4,4'-azobis(4-cyanovaleric acid) (0.02 eq with respect to the total monomer mole). Then, the reaction mixture was degassed by nitrogen gas for 30 minutes and immersed in a heated oil bath at 70 °C for 24 h. The polymer was precipitated in diethyl ether and the resulting residue collected by filtration and washed several times with diethyl ether. The monomer composition (%) was calculated by ^{1}H NMR, M_n ($g.mol^{-1}$) and $Đ$ were determined by SEC.

General procedure for aminolysis reaction

The reaction was adopted from previous literature.[3] The polymer (10 g) was dissolved in THF (500 mL). The solution was stirred and degassed for 30 minutes. Then, propylamine (20 eq with respect to **TLA** repeating units) was slowly added directly to the polymeric solution. The reaction was carried out at ambient temperature for 24 h. The solution was then concentrated by removing some solvent under reduced pressure, and the concentrated polymeric solution was precipitated in diethyl ether. The solid

was collected and filtrated by suction filtration and dried. The dried polymer was characterised by ^{1}H NMR, SEC, UV-Vis, and FT-IR techniques.

General procedure for the synthesis of poly(dimethyl acrylamide-monothiomaleimide)

The reaction was adopted from previous literature.[1] The polymer was dissolved in methanol (250 mL). Then, a solution of N-propylmonobromomaleimide (3 eq with respect to TLA repeating unit) and anhydrous sodium acetate (1 eq with respect to N-propylmonobromomaleimide) in 50 mL methanol was added to the polymeric solution. The reaction mixture was carried out for 10 minutes at room temperature. Eventually, the solution was concentrated and precipitated in diethyl ether. The brown solid was dissolved in THF and precipitated in diethyl ether three time, and filtrated by suction filtration. The solid was characterised by ^{1}H NMR, SEC, UV-Vis, and FT-IR techniques.

Photogelation of poly(dimethyl acrylamide-*stat*-monothiomaleimide)

The polymer (200 mg, 20 wt%) was dissolved in 1 mL DMF, then the solution was exposed to 365 nm Lumidox II 96-Position LED array (45 mW). Irradiation was carried out for 48 h.

Supporting Figures and Tables

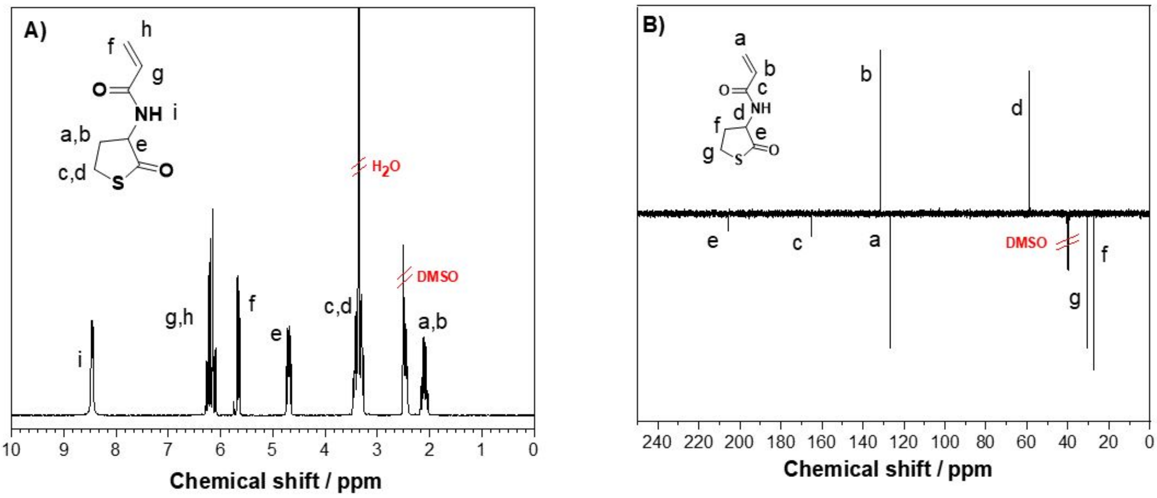

Fig S1. A) ^{1}H NMR of D,L-homocysteine thiolactone acrylamide (**TLA**) and B) ^{13}C NMR of **TLA**.

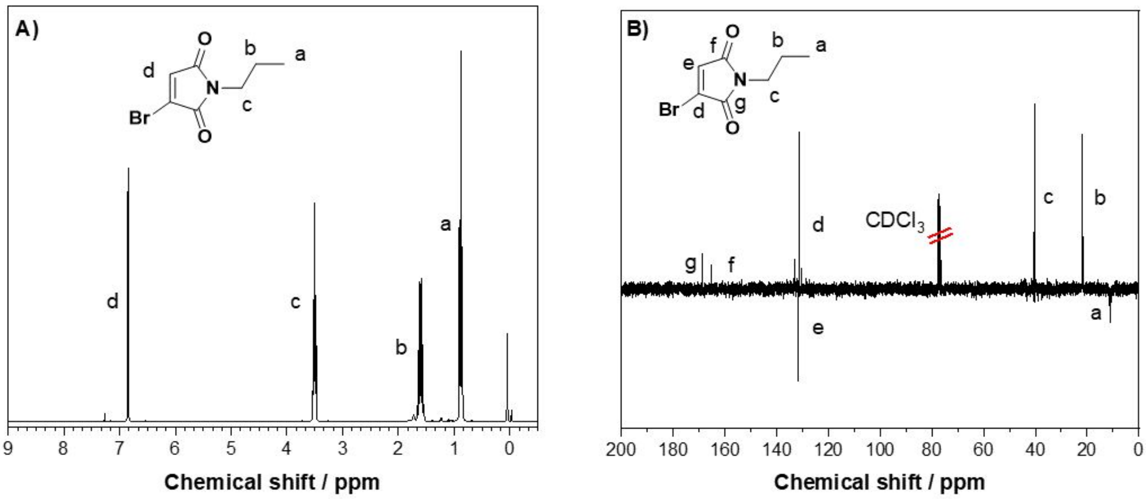

Fig S2. A) ^{1}H NMR of *N*-propyl-3-monobromomaleimide (MBM) and B) ^{13}C NMR of MBM measured in $CDCl_3$.

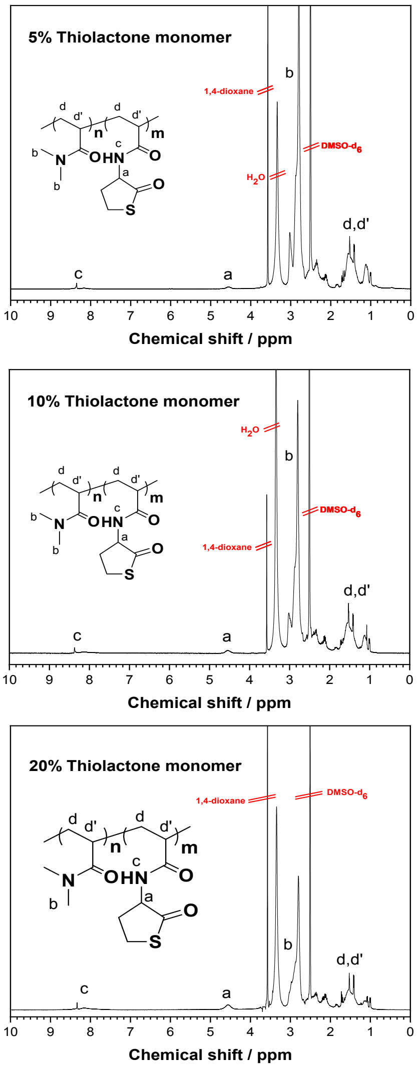

Fig S3. ^{1}H NMR of **poly(DMA-TLA)$_{5\%}$** (top), **poly(DMA-TLA)$_{10\%}$** (middle), and **poly(DMA-TLA)$_{20\%}$** (right) in DMSO-d_6 solvent.

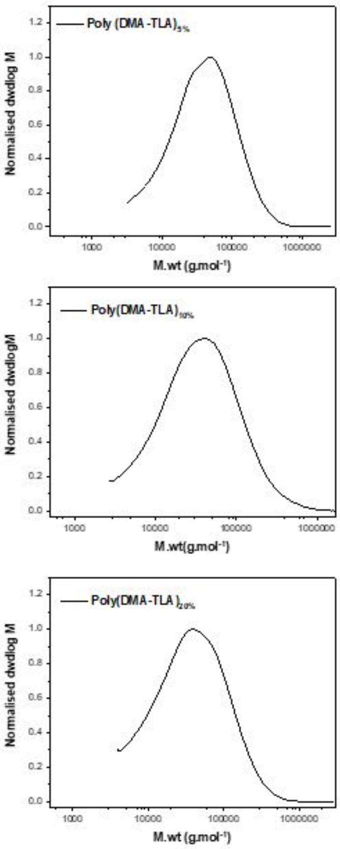

Fig S4: RI detector-SEC traces of **poly(DMA-TLA)$_{5\%}$** (top), **poly(DMA-TLA)$_{10\%}$** (middle), and **poly(DMA-TLA)$_{20\%}$** (bottom) in DMF eluent.

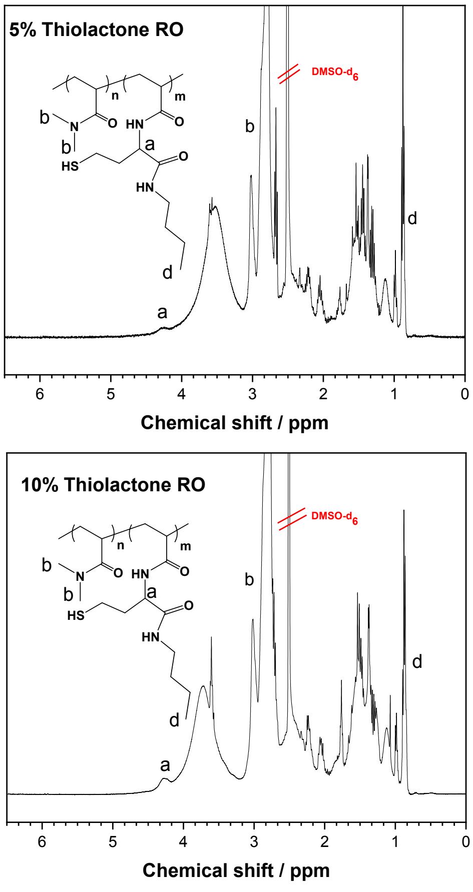

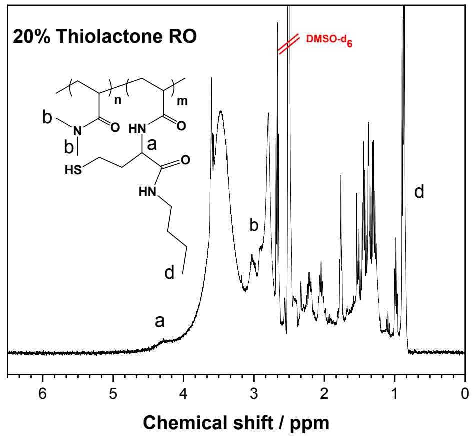

Fig S5: ^{1}H NMR of the ring-opening **poly(DMA-TLA)$_{5\%}$** (top), **poly(DMA-TLA)$_{10\%}$** (middle), and the ring opening **poly(DMA-TLA)$_{20\%}$** (bottom) in DMSO-d_6 solvent.

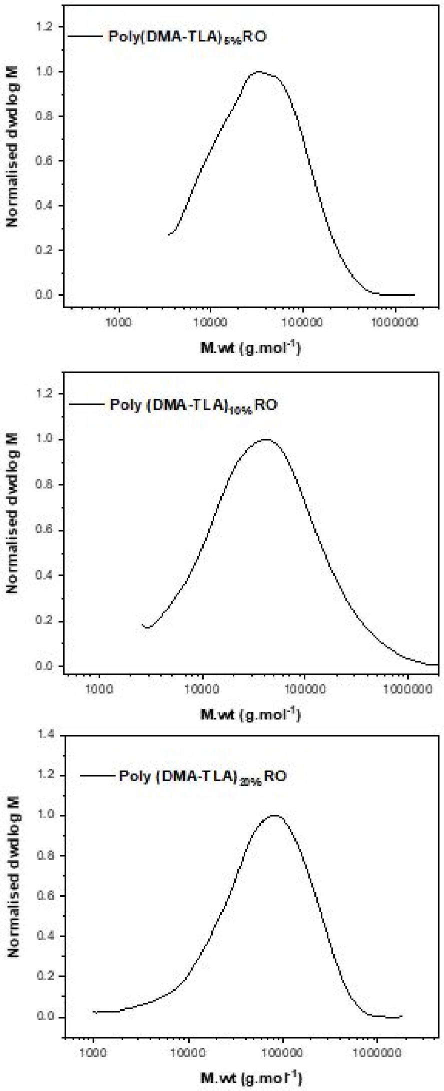

Fig S6. RI detector-SEC traces of the ring-opening **poly(DMA-TLA)$_{5\%}$** (top), **poly(DMA-TLA)$_{10\%}$** (middle), and **poly(DMA-TLA)$_{20\%}$** (bottom) after aminolysis reaction in DMF eluent.

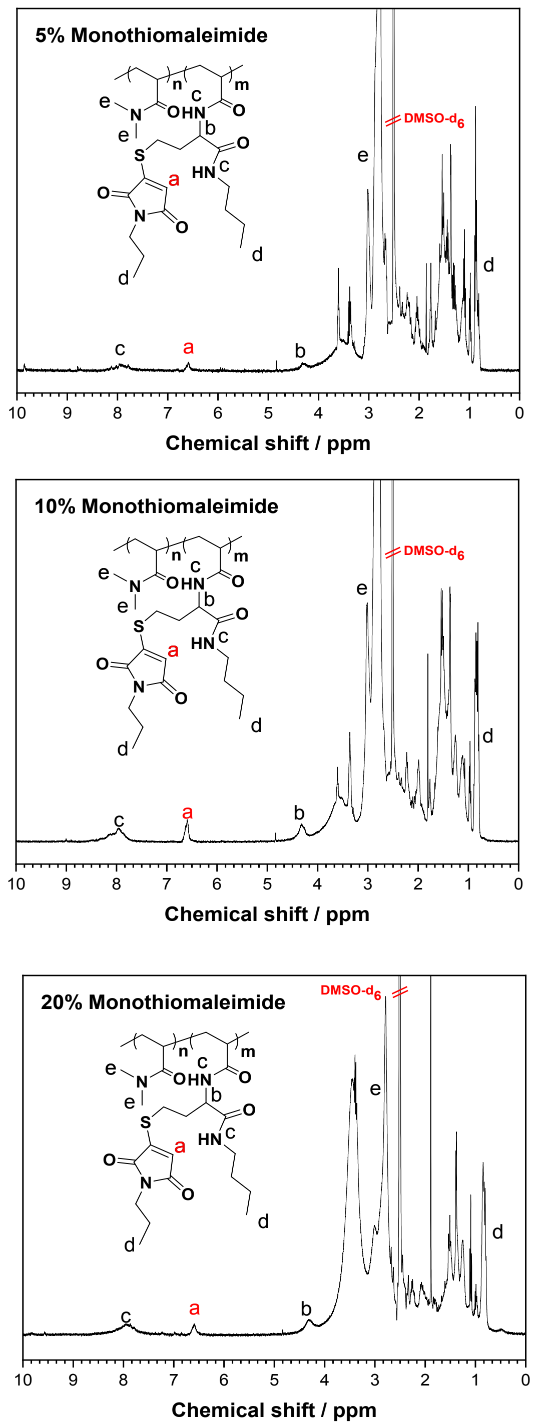

Fig S7: ^{1}H NMR of **poly(DMA-MTM)$_{5\%}$** (top), **poly(DMA-MTM)$_{10\%}$** (middle), and **poly(DMA-MTM)$_{20\%}$** (bottom) in DMSO-d_6 solvent.

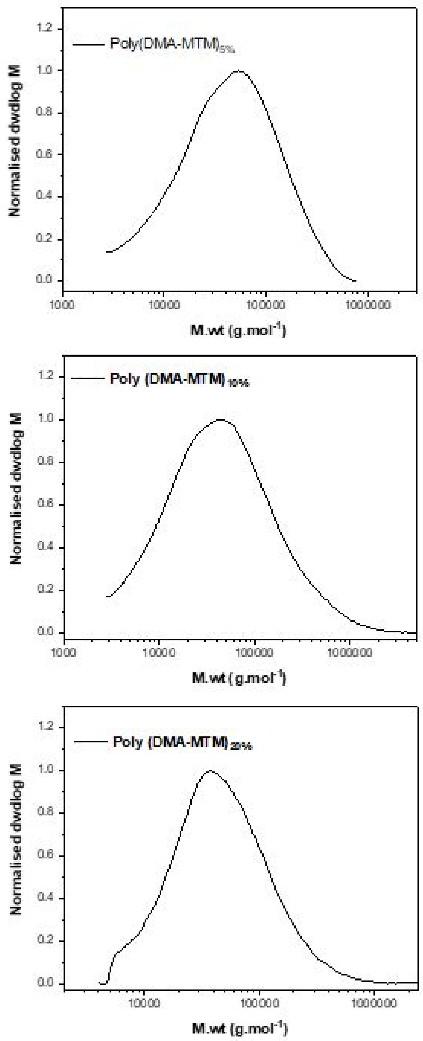

Fig S8: RI detector-SEC traces of **poly(DMA-MTM)$_{5\%}$** (top), **poly(DMA-MTM)$_{10\%}$** (middle), and **poly(DMA-MTM)$_{20\%}$** (bottom) in DMF eluent.

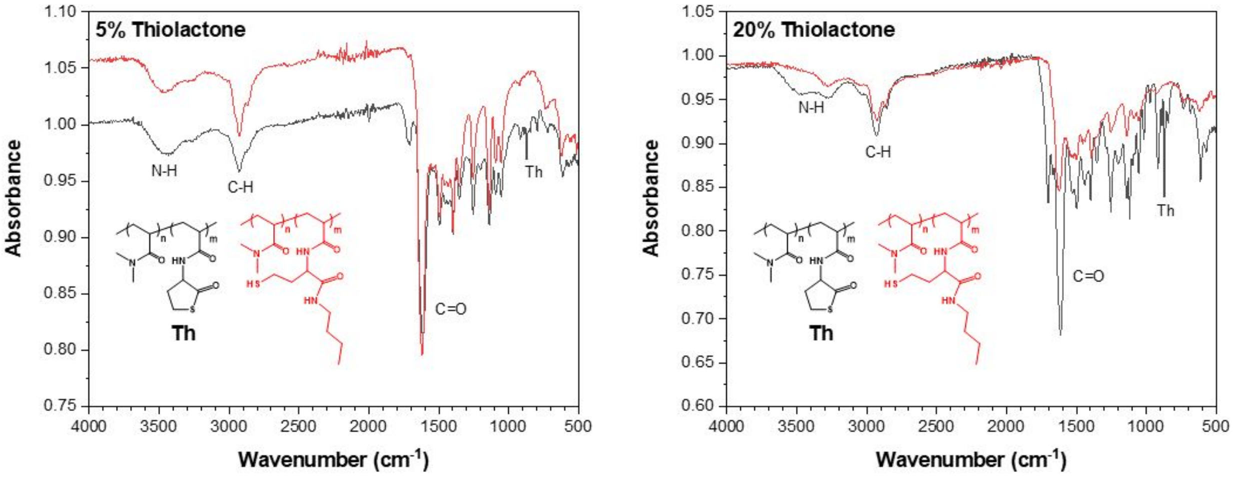

Fig S9: FT-IR spectrums of **poly(DMA-TLA)$_{5\%}$** and its ring-opening analogue (left) and **poly(DMA-TLA)$_{20\%}$** and its ring-opening analogue (right).

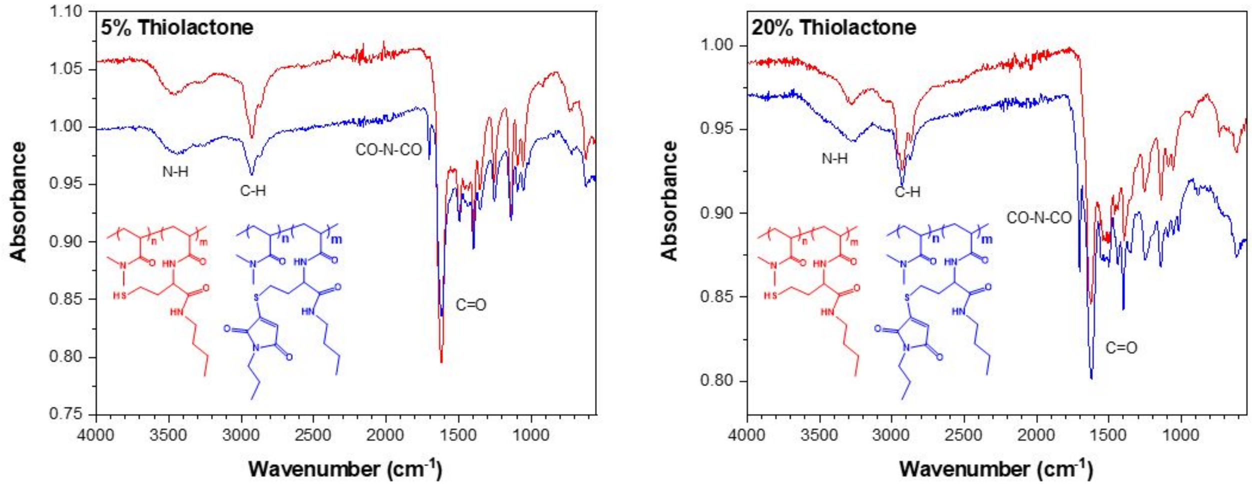

Fig S10: FT-IR spectrums of the ring-opening **poly(DMA-TLA)$_{5\%}$** and **poly(DMA-MTM)$_{5\%}$** (left) and the ring-opening **poly(DMA-TLA)$_{20\%}$** and **poly(DMA-MTM)$_{20\%}$** (right).

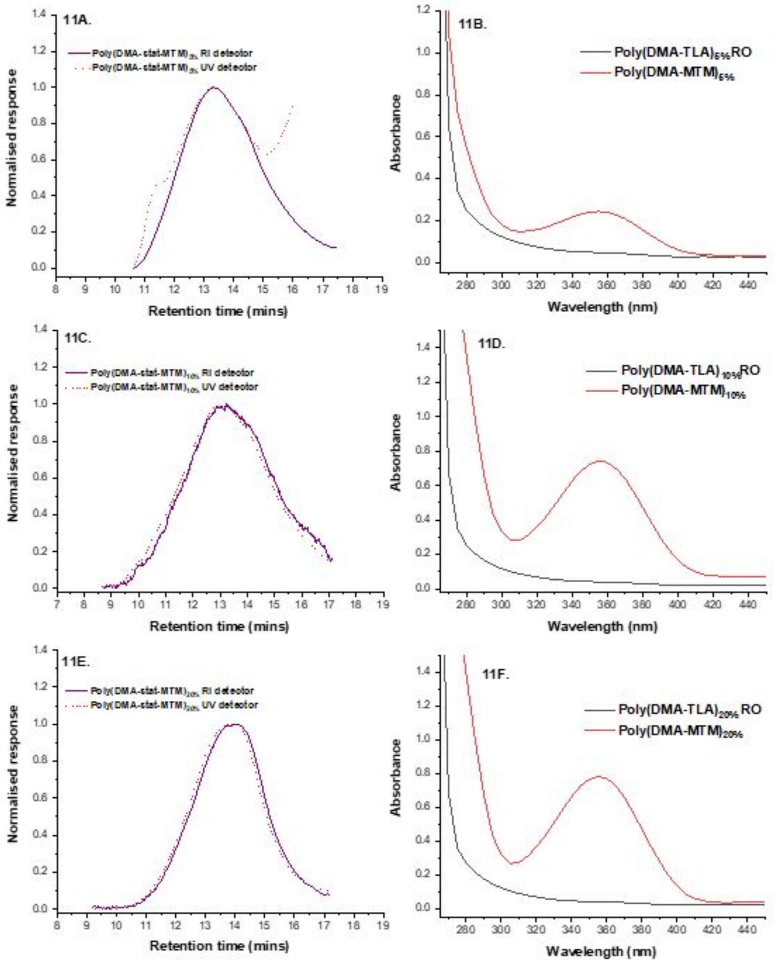

Fig S11. Coinciding RI and UV (λ = 365 nm) for **poly(DMA-MTM)$_{5\%}$** (A), **poly(DMA-MTM)$_{10\%}$** (C), and **poly(DMA-MTM)$_{20\%}$** (E); UV-Vis spectrums of **poly(DMA-MTM)$_{5\%}$** (B), **poly(DMA-MTM)$_{10\%}$** (D), and **poly(DMA-MTM)$_{20\%}$** (F) and their ring-opened polymer analogues.

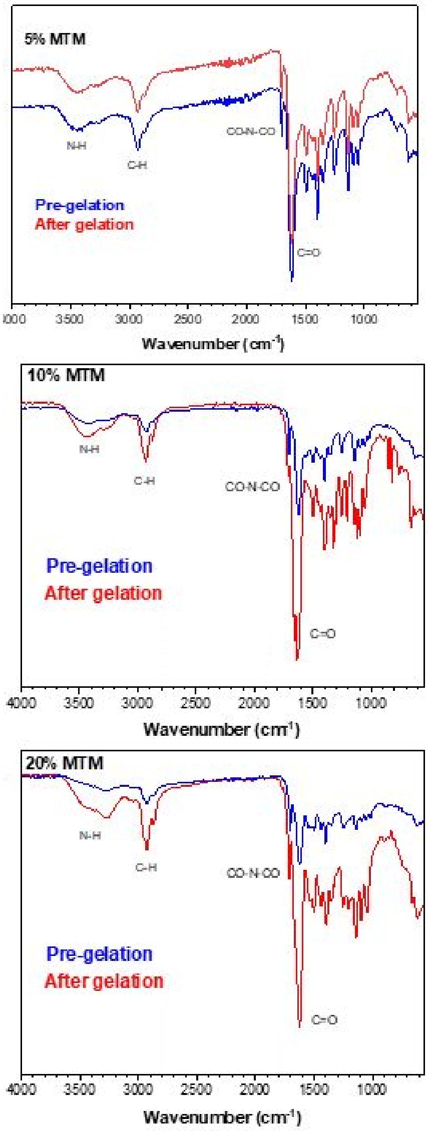

Fig S12: FT-IR spectra of dried **poly(DMA-MTM)$_{5\%}$** (top), **poly(DMA-MTM)$_{10\%}$** (middle), and **poly(DMA-MTM)$_{20\%}$** (bottom) before and after photo-gelation.

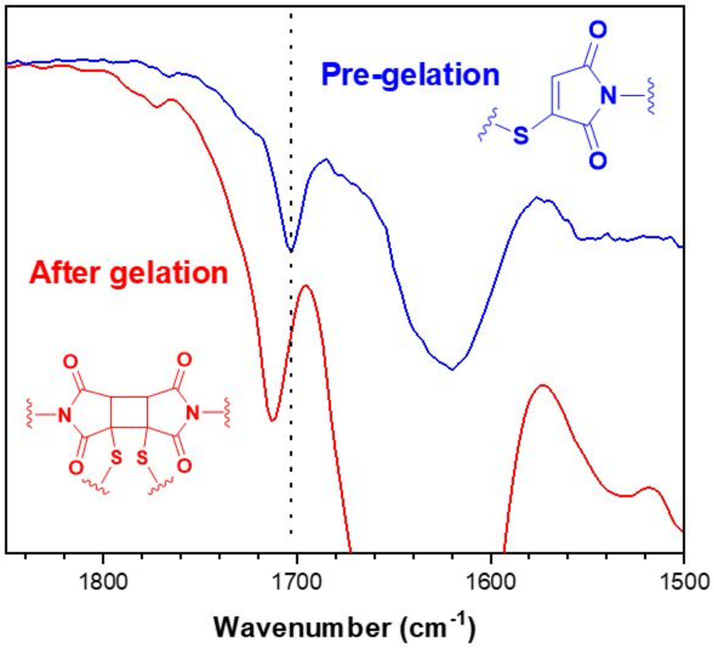

Fig S13: FT-IR spectrum of dried **poly(DMA-MTM)$_{10\%}$** at 1702 cm^{-1} and its shift to 1712 cm^{-1} due to the loss of conjugation of imide with C=C after photo-gelation.

Table S1. The summary of the results from the rheological measurement and compression test for **poly(DMA-MTM)s** gels.

Entry	Storage modulus (G') (Pa)	Loss modulus (G'') (Pa)	Crossover (γ %)	Stress at the breaking point	Strain at the breaking point (%)
**MTM$_{5\%}$**	903.154±159.65	144.48±25.54	-	2934.65±945.139 (kPa)	49.98±0.013
**MTM$_{10\%}$**	2807.69±334	64.53±14.70	71.2	326.81±1.3 (MPa)	2.67±0.17
**MTM$_{20\%}$**	6036.17±461.28	838.36±277.07	10.9	612.65±0.45 (MPa)	3.84±0.1

These values and the standard deviation were collected from three different measurements.

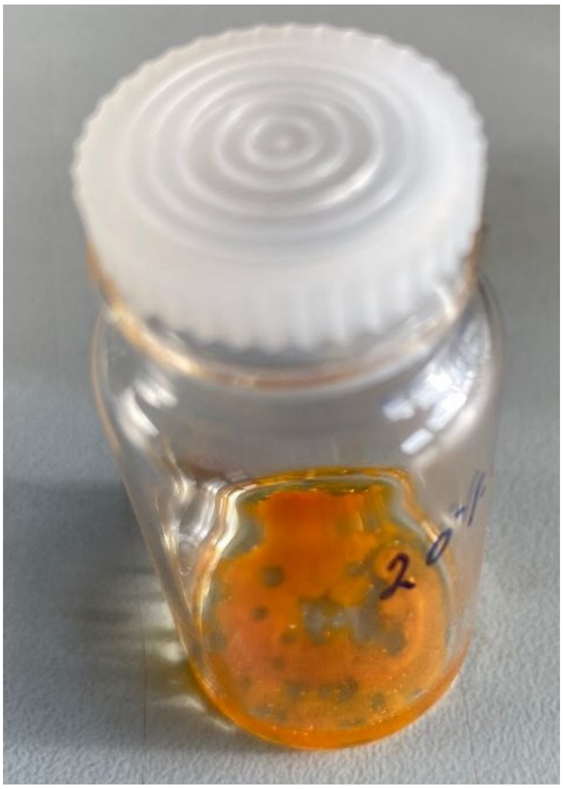

Fig S14: The insolubility of **poly(DMA-MTM)$_{20\%}$** in water at concentration 20 wt% before exposing the solution to UV light (365 nm).

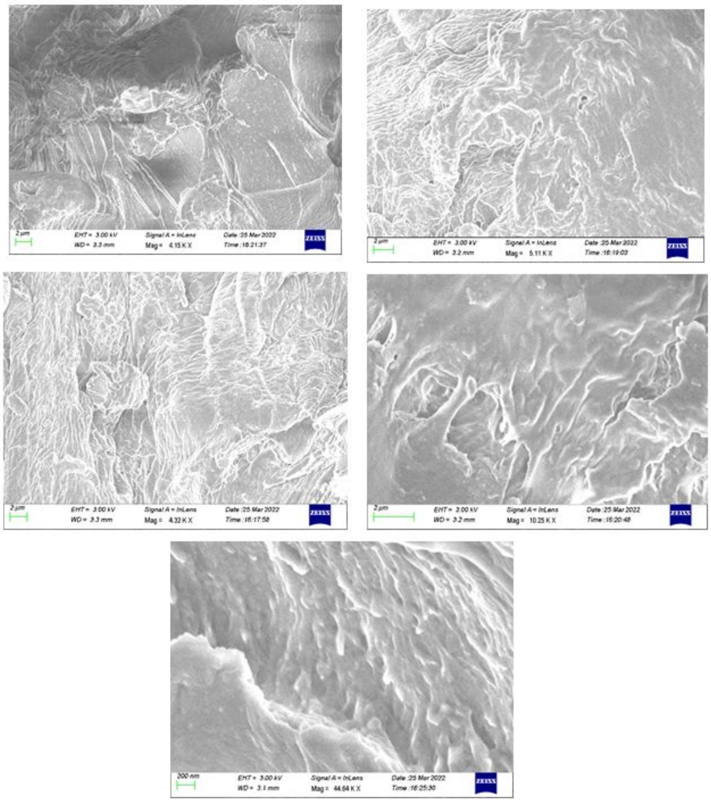

Fig S15: Scanning electron microscopy (SEM) images for the dried **poly(DMA-MTM)$_{5\%}$** network cured under UV light (365 nm) using DMF as a solvent at concentration (20 wt%).

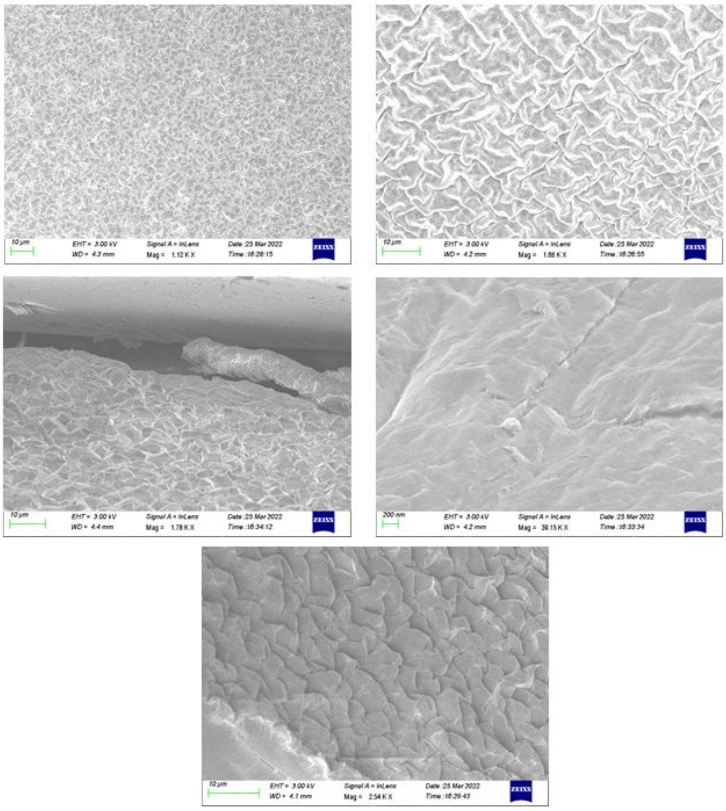

Fig S16: Scanning electron microscopy (SEM) images for the dried **poly(DMA-MTM)$_{10\%}$** network cured under UV light (365 nm) using DMF as a solvent at concentration (20 wt%).

References

1. Reese, C. M.; Thompson, B. J.; Logan, P. K.; Stafford, C. M.; Blanton, M.; Patton, D. L. Sequential and One-Pot Post-Polymerization Modification Reactions of Thiolactone-Containing Polymer Brushes. *Polym. Chem.* **2019**, *10*, 4935–4943.
2. Aljuaid, M.; Liarou, E.; Town, J.; Baker, J. R.; Haddleton, D. M.; Wilson, P. Synthesis and [2+2]-Photodimerisation of Monothiomaleimide Functionalised Linear and Brush-like Polymers. *Chem. Commun.* **2020**, *56*, 9545–9548.
3. Espeel, P.; Du Prez, F. E. One-Pot Multi-Step Reactions Based on Thiolactone Chemistry: A Powerful Synthetic Tool in Polymer Science. *Eur. Polym. J.* **2015**, *62*, 247–272.
